# Origin and timing of New Zealand's earliest domestic chickens: Polynesian commensals or European introductions?

**DOI:** 10.1098/rsos.160258

**Published:** 2016-08-03

**Authors:** Jamie R. Wood, Michael J. B. Herrera, R. Paul Scofield, Janet M. Wilmshurst

**Affiliations:** 1 Long-term Ecology Lab, Landcare Research, PO Box 69040, Lincoln 7640, New Zealand; 2 Australian Centre for Ancient DNA, School of Biological Sciences, University of Adelaide, North Terrace Campus, South Australia 5005, Australia; 3 Canterbury Museum, Rolleston Avenue, Christchurch 8013, New Zealand; 4 School of Environment, The University of Auckland, Private Bag 92019, Auckland 1142, New Zealand

**Keywords:** archaeology, commensal species, European-contact, Pacific, prehistory, radiocarbon dating

## Abstract

Human settlers transported chickens (*Gallus gallus domesticus*) to most East Polynesian archipelagos between AD 1000 and 1300; however, it has long been assumed that New Zealand was an exception. Despite the fact that chicken bones have been recovered from localities of early archaeological middens in New Zealand, their age and genetic relationships have never been critically assessed. Here, we test the assumption that chickens were not introduced to New Zealand during prehistory through ancient DNA and radiocarbon analyses of chicken bones from sites of Māori middens containing prehistoric material. The chickens belong to the widespread mitochondrial control region haplogroup E. Radiocarbon dating reveals that the bones are not prehistoric, but are still the earliest chicken remains known from New Zealand. Two of the bones pre-date permanent European settlement (*ca* 1803s onwards) but overlap with the arrival of James Cook's second voyage (1773–1774), and, therefore, they are likely to be chickens, or progeny thereof, liberated during that voyage. Our results support the idea that chickens were first introduced to New Zealand by Europeans, and provide new insights into Māori uptake and integration of resources introduced during the early post-European period.

## 1. Introduction

The colonization of the remote islands of East Polynesia from the AD eleventh to thirteenth centuries was the last major migration of modern humans to habitable lands [1,2]. During this dispersal, voyagers transported a variety of cultigens and commensal species throughout the East Polynesian archipelagos, including taro (*Colocasia esculenta*), bottle gourd (*Lagenaria siceraria*), paper mulberry (*Broussonetia papyrifera*), Pacific rat (*Rattus exulans*), pig (*Sus scrofa*) and chicken (*Gallus gallus domesticus*) [3–7]. Previous studies have demonstrated how the analysis of the distribution, morphology, genetics and age of these translocated species can provide important insights into the timing and origins of prehistoric movements of people throughout the Pacific (e.g. [1,8–15]). Prehistoric bones indicate that some commensal species, such as the Pacific rat, were nearly ubiquitous throughout East Polynesia [8,9]. However, the distribution of other species remains poorly resolved [3,6]. For example, prehistoric chicken bones have been excavated from early Polynesian middens on some of the most remote islands in the region, including Rapa Nui/Easter Island and Hawai'i [14], yet they remain conspicuously absent from the equally remote but cooler subtropic and temperate southern islands of Polynesia (including New Zealand, and the Chatham, Auckland, Kermadec and Norfolk Islands) [3,6]. The apparent absence of prehistoric chicken remains on these islands raises many questions [3]. For example, were chickens introduced by the first Polynesian settlers but then subsequently lost to disease, low propagule pressure, competition and predation or because there was no need to sustain domestic chickens in the presence of abundant large native flightless birds? Were they never taken in the first place [16–19]? Or, have chicken bones in prehistoric middens been overlooked as recent contamination? Answering these questions will help to resolve broader questions regarding the frequency and longevity of inter-island voyaging following initial settlement. This is of particular relevance on Pacific islands, where large prey species were hunted to extinction relatively quickly and human population growth meant food resources soon became limited. In such scenarios, returning to ancestral islands to source chickens (or other food resources) would clearly be desirable if long-distance inter-island travel was still feasible. For example, in Tonga, radiocarbon dating suggests that chickens were only introduced after the large native megapode (*Megapodius alimentum*) and iguana (*Brachylophus* sp.) had become extinct [3].

In the light of these long-debated and unresolved questions, it is perhaps surprising that the prehistoric presence of chickens in New Zealand has never been critically examined, especially given that the lack of evidence for their former absence is somewhat misleading. In fact, chicken bones have been excavated from several localities of prehistoric Polynesian (Māori) middens throughout New Zealand, including potentially archaic sites (e.g. [20–26]). Chicken bones in Māori middens are usually assumed to represent disturbance and incorporation of recent material into older layers (e.g. [26]). In some sites, this is likely, especially where stratigraphy is disturbed, and/or where other items, such as sheep (*Ovis aries*) bones or glass, are also present (e.g. [23,24]). However, such dogma creates the potential for genuine prehistoric chicken remains to be overlooked.

**Figure 1.**
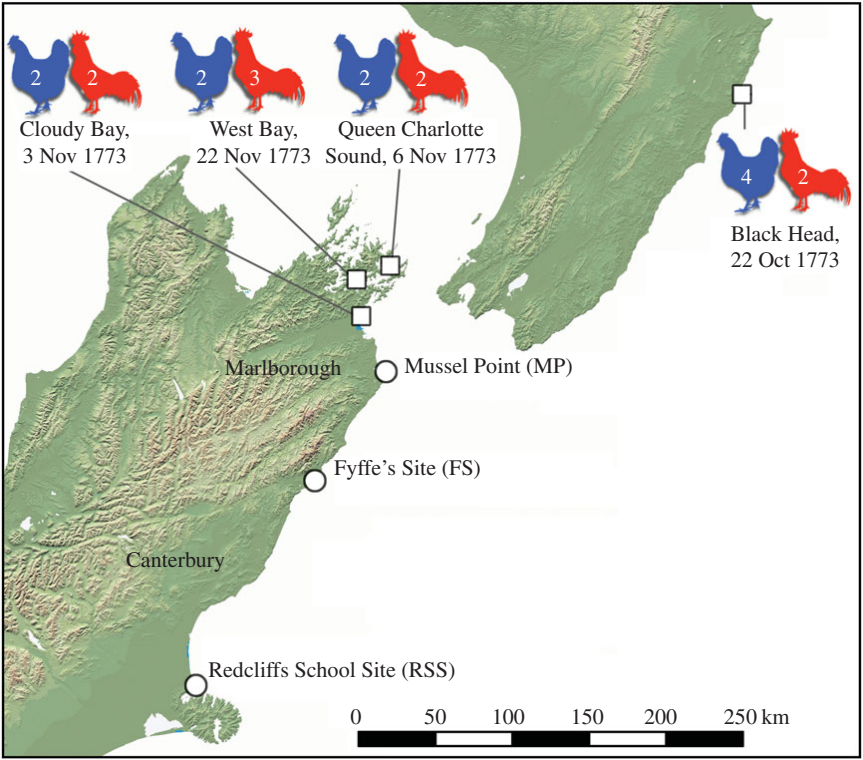
Locations (squares) and number of live chickens liberated or gifted to Māori tribes on Captain Cook's second voyage to New Zealand in 1773 (male red, female blue). The locations of the three chicken bones from Māori middens examined in this study are marked by circles.

Here, we provide the first radiocarbon and ancient DNA analyses of chicken bones from sites of Māori middens containing prehistoric material. We focus on bones from three different sites along the northeast coast of New Zealand's South Island (figure 1). The sites were selected as ideal candidates for finding potentially prehistoric chicken specimens, as they also contained pre-European faunal assemblages (electronic supplementary material), including bones of moa and other large birds (such as South Island adzebill (*Aptornis defossor*) and South Island goose (*Cnemiornis calcitrans*)) that became extinct within 200 years of initial human settlement [27–29].

## 2. Results

### 2.1. Radiocarbon dating

**Figure 2.**
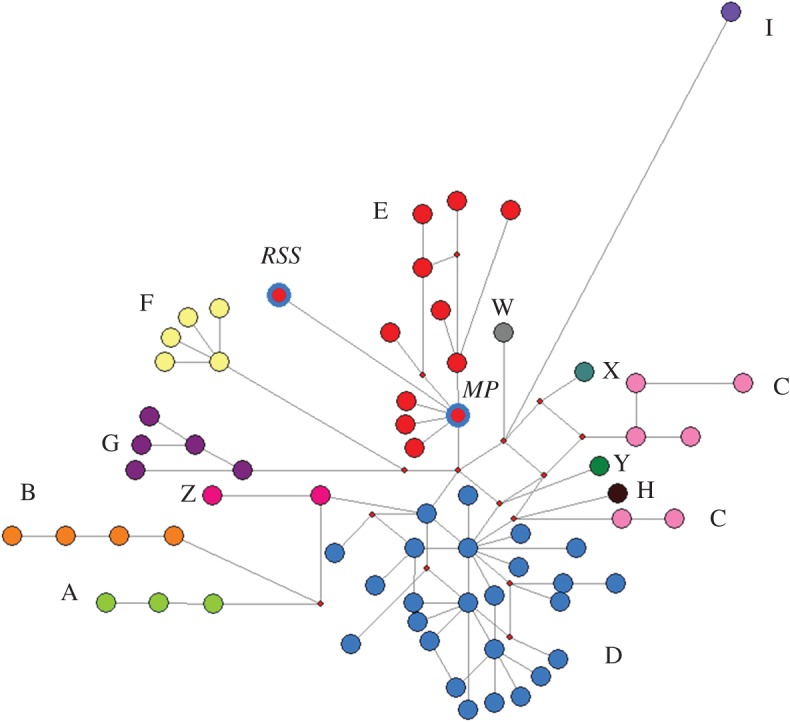
Haplotype network for chickens based on a 200 bp sequence of mitochondrial control region. Both the Redcliffs School Site (RSS) and Mussel Point (MP) specimens are within the widespread haplogroup E.

Gelatin fractions from the three bones studied fell within the required mass range for standard accelerator mass spectrometry (AMS) dating. The oldest bone was from Redcliffs School Site (RSS), returning an age of 226 ± 25 14C years before present (BP) (median age = AD 1756; 95.4% calibrated confidence range = AD 1650–1805; table 1 and figure 2). The bone from Mussel Point (MP) was only slightly younger at 211 ± 23 14C years BP (median age = AD 1757; 92.6% calibrated confidence range = AD 1652–1810; table 1 and figure 2). The bone from Fyffe's Site (FS) was dated at 164 ± 23 14C years BP (median age = AD 1840; 95.4% calibrated confidence range = AD 1675–1950; table 1 and figure 2).

**Table 1.** Radiocarbon dates for archaeological chicken bones from South Island, New Zealand. Wk, Waikato Radiocarbon Dating Laboratory code; CM, Canterbury Museum; CRA, conventional Radiocarbon Age. Owing to small sample sizes, d13C was measured on prepared graphite using the AMS spectrometer. The radiocarbon dates have therefore been corrected for isotopic fractionation. All isotope values measured on bone gelatin.

Wk number	CM number	site	powdered mass (g)	gelatin yield (%)	d^15^N	C:N	CRA	error	1 sigma calibrated ranges	2 sigma calibrated ranges
Wk38085	Av33059	Fyffe's Site, Kaikoura	0.18	7.5	7.66	3.37	164	23	AD 1684–1711 (16.2%)AD 1719–1730 (5.9%)AD 1803–1813 (5.4%)AD 1837–1883 (24.2%)AD 1925 – (16.5%)	AD 1675–1738 (29.8%)AD 1797–1950 (65.6%)
Wk38086	Av23181	Redcliffs School Site, Christchurch	0.22	4.1	6.73	3.36	226	25	AD 1666–1675 (8.3%)AD 1739–1797 (59.9%)	AD 1650–1690 (23.4%)AD 1726–1805 (72.0%)
Wk38088	Av17724	Mussel Point, Cape Campbell	0.2	9.5	8.04	3.39	211	23	AD 1670–1679 (8.9%)AD 1733–1785 (53%)AD 1794–1801 (6.4%)	AD 1652–1700 (22.3%)AD 1721–1810 (70.3%)AD 1836–1844 (0.9%)AD 1866–1878 (1.5%)AD 1932–1938 (0.5%)

### 2.2. Ancient DNA

Ancient DNA (200–201 bp of mitochondrial control region) was amplified and sequenced from the two oldest chicken bones (from RSS and MP), but was not obtained from the younger bone (from FS). The two sequences (Genbank accession nos. KX394444 and KX394445) varied by four nucleotides, all being G–A or C–T transitions. Both sequences fall within the widespread chicken mitochondrial control region haplogroup E (figure 2). The sequence from the RSS bone represents a previously unsequenced haplotype (confirmed by nearest BLAST match having 99% identity), while the sequence from the MP bone represents a widespread haplotype previously reported in chickens from Africa, Asia, Europe, South America and the Pacific.

## 3. Discussion

### 3.1. Age and origin of the chickens

The calibrated age ranges of the radiocarbon-dated chicken bones from two sites (RSS and MP) suggest they pre-date permanent European settlement (1830s). Although there are multiple probability distributions for the calibrated dates, the highest probabilities on the age ranges for these sites (table 1) occur before the 1830s, with the median age of *ca* AD 1760, meaning these are the earliest chicken remains known from New Zealand. The bone from FS is somewhat younger, although still relatively early (median age of AD 1840), yet the major probability distributions are bimodal, split between AD 1837 and 1883 (1 sigma). There is no significant difference between the ages of the three bones (test statistic *T* = 3.75; *χ*^2^ (0.5) = 5.99; 2 d.f.).

The potential for marine carbon to skew the radiocarbon age of archaeological chicken bones has previously been examined with regard to pre-Columbian chicken bones from Chile [11,14,30–34]. Owing to the small sample sizes, the d13C from the New Zealand chicken bones was measured on prepared graphite. However, all three bones reported here had d15N values (table 1) consistent with an entirely terrestrial diet (F. Petchey 2014, personal communication), and the radiocarbon dates are, therefore, unlikely to have been influenced by the marine reservoir effect. Although the accuracy of the dates appears to be robust, the precision of the dates is affected by a plateau in the radiocarbon calibration curve after *ca* AD 1800, meaning that there are wide confidence intervals around calibrated ages within this time period. However, the bones from RSS and MP are just old enough so as not to be affected by this plateau to the same degree as the FS specimen. The confidence ranges for the ages of the two older bones (more than 90% before 1810) appear to rule out early European settlers (who began arriving *en masse* from the 1830s onwards) as a source. However, as we discuss below, several other transient European visitors were visiting the New Zealand coastline prior to the 1830s, and their potential as sources for the chickens requires critical assessment.

The first of the early European (pre-1830) visitors to New Zealand were sealing gangs, who visited and explored the coastal areas of New Zealand in the late eighteenth and early nineteenth centuries. However, the calibrated age ranges for the RSS and MP chicken bones, together with what is known about the timing and location of sealing expeditions to New Zealand [35], appear to rule out sealers as a likely source. Only two sealing voyages to New Zealand can be confirmed prior to the main period of sealing between 1803 and 1840 [35]. The earliest phase of sealing (1803–1807) was concentrated along the Fiordland and Stewart Island coastlines in the far south of the South Island [35], and there is no evidence that sealing took place along the eastern South Island coastline north of Dunedin (the location of our chicken bones) at this time. Although there were some port visits by sealers in Canterbury (including Banks Peninsula) from 1809 to 1829 [35], these visits are too late to represent a likely source for the RSS and MP chicken bones.

Whalers also operated around the New Zealand coastline from the late eighteenth century, but can be ruled out as a likely source of the chickens. Most early whaling was concentrated around the northern North Island (e.g. Bay of Islands) [36]. Whalers do not appear to have made cruises down the South Island until the 1830s, with visits to Banks Peninsula from 1835 onwards [37].

**Figure 3.**
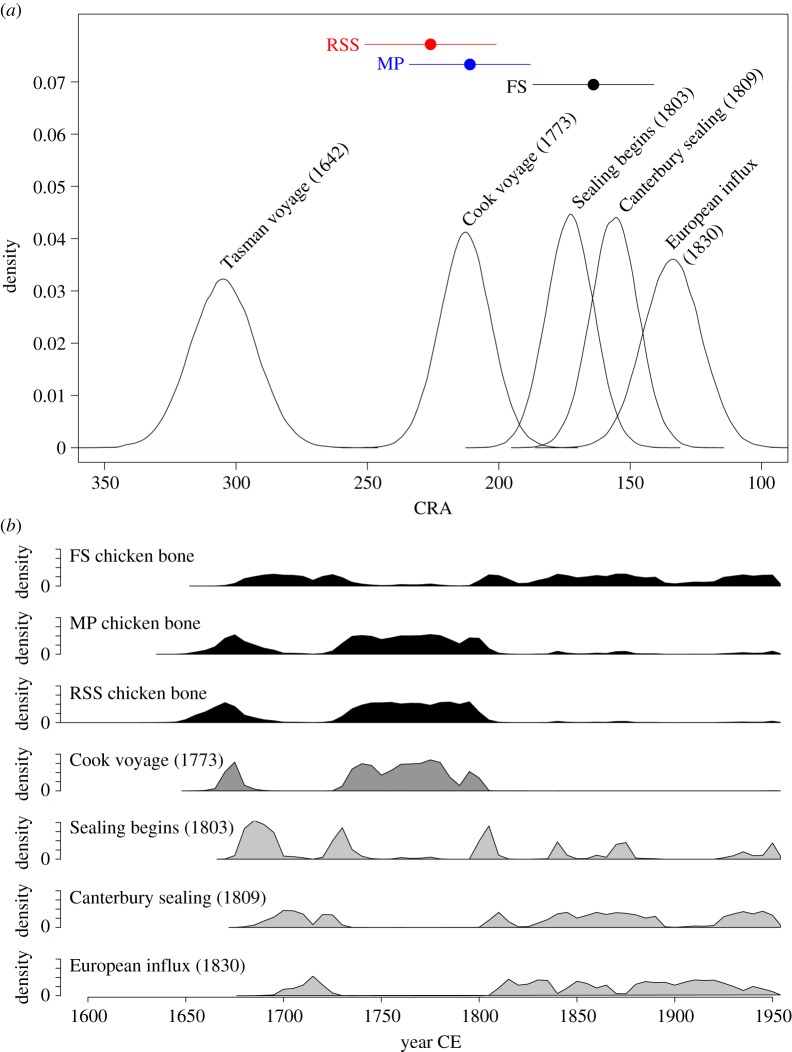
(*a*) Median and 2σ ranges for conventional radiocarbon ages (CRA) of chicken bones from Redcliffs School Site (RSS), Mussel Point (MP) and Fyffe's Site (FS), South Island, New Zealand, compared with distributions of the CRAs corresponding to calendar years for key events in the early European-contact period; (*b*) calibrated age distributions for New Zealand chicken bones compared with those for CRAs relating to key events in the early European-contact period.

Several cruises were made around the New Zealand coastline by exploration expeditions between the time of New Zealand's discovery by Europeans (Abel Tasman in 1642) and the beginning of sealing and whaling in the late eighteenth to early nineteenth centuries (e.g. Vancouver in 1791, Malaspina in 1793). However, there is no evidence to suggest that chickens were released onshore during these voyages [38]. Other early, yet unrecorded, expeditions may have shipwrecked upon the New Zealand coast, with the potential for live cargo to have escaped ashore [39]. However, just one expedition provides clear evidence for chickens having been liberated in New Zealand, with dates that coincide with our earliest dated specimens: that of Captain James Cook's second voyage to New Zealand in 1773. Therefore, in the light of the evidence outlined above, we suggest that the ages of the RSS and MP chicken bones are most consistent with them representing chickens, or near descendants thereof, brought to New Zealand on Cook's 1773 expedition (figures 2 and 3).

DNA sequences from both the RSS and MP chicken bones were from the globally distributed E-haplogroup, and so did not provide any information on the potential place of origin. The chickens released by Cook's expedition in New Zealand may have had mixed origins, as they definitely acquired chickens during a stop at the Cape Verde Islands ([40], p. 27), but may also have had chickens sourced from England, South Africa (during a stop there en route to New Zealand) and Tahiti/Tonga (chickens were not liberated in New Zealand until the expedition had returned from these islands).

#### 3.1.1. Ethnographic evidence for early uptake of introduced chickens by Māori

The occurrence of chicken bones in Māori middens from the South Island that date from before the main period of European settlement (after which chickens could be widely sourced) provides new insights into the adoption, spread and use of novel species by Māori during the early post-European-contact era. The fate of Captain Cook's chickens, or how long they may have persisted, has never been known. Cook himself appears to have felt that the chickens had slim chances of survival. For example, on 3 November 1773 he gave two cocks and two hens to a local of the Cloudy Bay/Port Underwood area, noting that these ‘he received with such indifferency, as gave me little hopes that proper care would be taken of them’ ([40], p. 285). Cook also recorded that upon gifting two cocks and four hens to a Māori chief near Black Head, south of Cape Kidnappers, on 22 October 1773 the chief appeared unimpressed with the birds ([40], p. 279). From these records, it might seem unlikely that Cook's chickens would have persisted long enough to form self-sustaining populations. However, there were some instances where it seems that they did. For example, Cook liberated chickens at West Bay in the Marlborough Sounds, northern South Island, in 1773, and upon returning the following year a fresh hen's egg was discovered in the forest, suggesting at least one of the females had survived ([40], p. 291 and 573). It appears that a large number of chickens were also liberated by Tobias Furneaux (Captain of *Adventure*, the second ship on Cook's second voyage) in the Marlborough Sounds, although the exact details of these were not recorded. Of them, Cook stated ‘More Cocks and Hens are left behind than I know of as several of our people had of these as well as my self, some of which they put on shore and others they sold to the Natives, whom we found took care enough of them’ ([40], p. 296).

**Figure 4.**
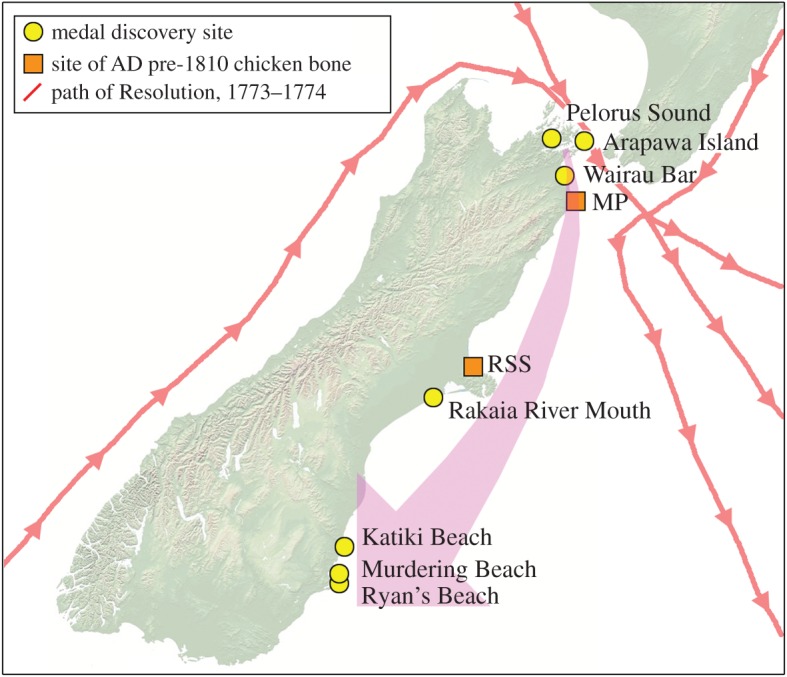
The path of Captain Cook's ship *Resolution* through New Zealand waters on his second voyage (1773–1774), with locations where medals associated with this voyage have been found, and where the two chicken bones in this study that pre-dated AD 1810 were excavated (MP, Mussel Point; RSS, Redcliffs School Site).

Previously, there has been no record of what became of the chickens Cook left in New Zealand, yet the bones examined here suggest they may have bred and been transported by Māori moving along the east coast of the South Island, and were possibly traded between groups. Further evidence supporting mobility of Māori and transport of European goods along this coast during the early post-contact era comes from the discovery locations of medals that were gifted by Cook to Māori living in the Marlborough Sounds, also on his second voyage. At least a dozen of these medals have since been discovered in association with sites of Māori settlement, mainly in the Marlborough Sounds and along the east coast of the South Island [41,42] (figure 4).

#### 3.1.2. Significance of historic introduction of chicken

The apparent rapid uptake of chickens by Māori in the late eighteenth century and their dispersal around New Zealand has parallels with a number of other food items introduced by early European visitors to New Zealand, such as white potatoes, turnips, carrots and cabbages [43,44]. Māori were quick to realize the potential of these new resources [17] and rapidly integrated them into their livelihoods, often resulting in significant changes to their society and economy [17,43,44]. This rapid uptake and dispersal of new food commodities is consistent with the idea that food resources were limited for Māori populations during the initial European-contact era, and had been so for several centuries prior to this time [17].

At the time of initial settlement in the AD thirteenth century [1], New Zealand's terrestrial fauna was dominated by large, flightless and naive birds, and abundant land-breeding seabirds and pinnipeds, which provided a rich source of easily hunted animal protein that helped to sustain early population growth [17]. However, in less than two centuries, intense hunting pressure [27] and forest clearance by fire [45–47] caused massive and widespread extinctions and a reduction in large easily harvested prey species. This in turn led to changes to Māori society (e.g. increased warfare) and economy (e.g. construction of fortified villages and cidatels) [17]. If chickens had not been introduced or successfully established in New Zealand at the time of initial settlement, it is reasonable to assume that by AD 1450 (when extinction of megafauna occurred and other protein sources were in decline), that given the ability, Māori would have made return voyages to their ancestral Polynesian islands to source chickens (and other protein resources such as pigs) [3,17]. The nearest archipelagos to New Zealand that had chickens (and pigs) at this time were the southern Cook Islands [6], only several weeks of open-ocean voyaging away to the northeast [48]. However, the New Zealand archaeological record lacks any evidence for material cultural exchange with East Polynesia after *ca* AD 1450, suggesting long-distance inter-island voyaging had ceased by this time. This concurs with the finding more generally in East Polynesia for a marked decline in long-distance voyaging and inter-island exchanges after AD 1450 [49].

Chicken bones are relatively rare in pre-1830s New Zealand archaeological middens, and the chicken bones from the three sites we selected for our analyses represent a significant proportion of the total chicken remains known from such sites. For this reason, and the fact that the chicken bones were from sites that also contained bones of large bird species that went extinct in the first 200 years after initial settlement, we targeted our analyses towards specimens offering the greatest likelihood of being prehistoric chickens. Although the dates for the chicken bones make them the oldest known chicken remains from New Zealand, the fact they are all eighteenth century or younger strongly suggests that chickens were either not introduced prehistorically, or if they were, they did not persist long enough to leave a trace in the archaeological record. If chickens had been established in New Zealand by the first settlers, we believe chicken bones would be more abundant and widespread in middens throughout the country, as they tend to be on other Polynesian islands where they were introduced and successfully established during prehistory [3]. Although it is speculative and difficult to prove, it seems probable that when the first Polynesians arrived in New Zealand and found an abundance of large, easily hunted, flightless birds, efforts to establish the domestic chicken, which required a certain amount of husbandry, may have been rapidly abandoned. The same explanation may apply to other Pacific archipelagos with apparent anomalous absences of prehistoric commensals [17]. For example, in New Caledonia, pigs were not introduced prehistorically, despite the relatively close proximity to source islands (Vanuatu) [3]. As with New Zealand, the first settlers to New Caledonia would have encountered large animals, including giant bats, turtles and flightless birds such as *Sylviornis neocaledoniae*, which would have provided easy hunting in the initial settlement period.

## 4. Conclusion

We have radiocarbon dated and sequenced DNA from the three most likely candidates for prehistoric chicken bones from New Zealand. Our results demonstrate that all were deposited during the early European-contact era. This finding strongly supports the hypotheses that chickens were either not brought to New Zealand by the first Polynesian settlers, or did not persist for very long following their introduction, due to the abundance of large native animals that could easily be hunted. However, sources of large native animals had become depleted by the time Captain Cook introduced chickens in 1773, after which chickens became readily integrated into Māori livelihoods, and were moved around New Zealand by Māori at this time. The fact that chickens (and other commensals such as pigs) were not introduced to New Zealand soon after the AD fifteenth century extinction of megafauna, such as occurred in Tonga, provides compelling evidence for the idea that Māori had lost the ability/knowledge for long-distance inter-island voyaging by this time.

## 5. Methods

### 5.1. Sites and samples

Chicken bones from three Māori midden deposits were selected for study: RSS, Christchurch (NZ Archaeological Association site number M36/24); Mussel Point, Marlborough (NZ Archaeological Association site number Q29/1) and Fyffe's site, Avoca Point, Kaikoura (NZ Archaeological Association site number S49/46). Although the bones had been collected some decades prior to this study, and there were no exact details on stratigraphic contexts available, faunal remains, artefacts and radiocarbon dates from the same localities show that each of the sites included occupation layers from the earliest phase of human settlement. Faunal assemblages from these sites also include significant proportions of bird species that became extinct prior to European settlement (electronic supplementary material, figure S1). Archaeological sites on Redcliffs Flat (which includes the RSS) span from the earliest phase of human settlement in New Zealand to recent times [23]. Further details of the study sites are presented in the electronic supplementary material.

### 5.2. Radiocarbon dating

Subsamples of chicken bones were submitted to the Waikato Radiocarbon Dating Laboratory in New Zealand for analysis. The samples were cleaned and ground, decalcified in 2% HCl, rinsed and dried. The remaining fraction was gelatinized in HCl (pH 3.0) at 90°C for 4 h before being ultrafiltered and freeze-dried, and dated using accelerator mass spectrometry (AMS). Radiocarbon dates were calibrated using the ShCal13 curve [50] via OxCal 4.2 [51].

### 5.3. Ancient DNA

Ancient DNA extractions and the setting up of PCR reactions were performed in a dedicated, purpose-built ancient DNA facility at Landcare Research, Lincoln, New Zealand. DNA was extracted from 22 to 31 mg samples of bone by incubation overnight at 55°C in 945 µl of 0.5 M EDTA (pH 8.0), 20 µl of 10% SDS and 35 µl of 20 mg ml^−1^ Proteinase-K, followed by extraction of the supernatant using the DNeasy Blood & Tissue Kit (Qiagen). Two microlitres of carrier RNA were added along with the AL buffer. A variable and globally well-characterized fragment of the chicken mitochondrial control region was amplified using the primers GG144 (5′-ACCCATTATATGTATACGGGCATTAA-3′) and GG387 (5′-CGAGCATAACCAAATGGGTTAGA-3′) [14]. PCR reactions (final volume 25 µl) contained 1 mg ml^−1^ bovine serum albumin, 1× PCR buffer, 2 mM MgSO_4_, 200 µM dNTP, 1 µM each primer, 1 U Platinum HiFi Taq (Invitrogen), 0.5 U shrimp DNase (Affymetrix) and 2 µl of DNA extract. The PCR master mix was incubated at 37°C for 15 min and 65°C for 15 min, prior to addition of the DNA template to allow the shrimp DNase to digest double-stranded DNA contaminants in the reagents. This was an essential step, as chicken DNA is a known contaminant of some PCR reagents, and can result in the amplification of non-endogenous products when working with ancient chicken DNA extracts [14,52]. PCR products were visualized on a 3% agarose gel, and amplified products were purified using ExoSAP-IT (Affymetrix) following the manufacturer's protocol, and bidirectionally sequenced using BigDye terminator technology on a capillary sequencer. The RSS and MP sequences were aligned with available whole mitochondrial genomes representing all-known chicken mitochondrial haplogroups (electronic supplementary material, table S1) using MUSCLE algorithm in GENIOUS v. 7.1.2. The alignment was truncated to 200 bp corresponding to the ancient DNA fragment. To identify the haplogroup assignment of the NZ sequences, a phylogenetic network was estimated via median-joining network [53] using NETWORK v. 4.6.1 (from fluxus-engineering.com). Following this, the RSS and MP sequences were aligned to a dataset comprising chicken mitochondrial control region sequences with known haplogroup and geographical assignments.

## Supplementary Material

Supporting information (text file): description of study sites and rationale for sampling

## Supplementary Material

Revised_Supp_Info_no mark up

## Supplementary Material

SI Table 1: Mitochondrial control region haplotypes for chickens (results of network analysis)

## Data accessibility

DNA sequences from the New Zealand chicken bones are available from GenBank (accession nos. KX394444 and KX394445). All other data are presented in the paper or electronic supplementary material.

## References

[1] Wilmshurst JM, Anderson AJ, Higham TFG, Worthy TH 2008 Dating the late prehistoric dispersal of Polynesians to New Zealand using the commensal Pacific rat. Proc. Natl Acad. Sci. USA 105, 7676–7680. (doi:10.1073/pnas.0801507105) 18523023 10.1073/pnas.0801507105 PMC2409139

[2] Wilmshurst JM, Hunt TL, Lipo CP, Anderson AJ 2011 High-precision radiocarbon dating shows recent and rapid initial human colonization of East Polynesia. Proc. Natl Acad. Sci. USA 108, 1815–1820. (doi:10.1073/pnas.1015876108) 21187404 10.1073/pnas.1015876108 PMC3033267

[3] Anderson A 2009 The rat and the octopus: initial human colonization and the prehistoric introduction of domestic animals to remote Oceania. Biol. Invas. 11, 1503–1519. (doi:10.1007/s10530-008-9403-2)

[4] Whistler WA 1991 Polynesian plant introductions. In Islands, plants, and Polynesians (eds Cox PA, Banack SA), pp. 41–66. Portland, OR: Dioscorides Press.

[5] Prebble M 2008 No fruit on that beautiful shore: what plants were introduced to the subtropical Polynesian islands prior to European contact. Terra Australis 29, 227–251.

[6] Storey AA, Ladefoged T, Matisoo-Smith EA 2008 Counting your chickens: density and distribution of chicken remains in archaeological sites of Oceania. Int. J. Osteoarchaeol. 18, 240–261. (doi:10.1002/oa.947)

[7] Kirch PV 1982 Ecology and the adaptation of Polynesian agricultural systems. Archaeol. Oceania 17, 1–6. (doi:10.1002/j.1834-4453.1982.tb00032.x)

[8] Matisoo-Smith E 1994 The human colonisation of Polynesia. A novel approach: genetic analyses of the Polynesian rat (*Rattus exulans*). J. Polynesian Soc. 103, 75–87.

[9] Matisoo-Smith E 2007 Animal translocations, genetic variation, and the human settlement of the Pacific. In Genes, language, & culture history in the southwest Pacific (ed. Friedlaender JS), pp. 157–170. Oxford, UK: Oxford University Press.

[10] Larson G et al. 2007 Phylogeny and ancient DNA of *Sus* provides insights into neolithic expansion in Island Southeast Asia and Oceania. Proc. Natl Acad. Sci. USA 104, 4834–4839. (doi:10.1073/pnas.0607753104) 17360400 10.1073/pnas.0607753104 PMC1829225

[11] Gongora J et al. 2008 Indo-European and Asian origins for Chilean and Pacific chickens revealed by mtDNA. Proc. Natl Acad. Sci. USA 105, 10 308–10 313. (doi:10.1073/pnas.0801991105) 10.1073/pnas.0801991105 PMC2492461 18663216

[12] Oskarsson MCR, Klutsch CFC, Boonyaprakob U, Wilton A, Tanabe Y, Savolainen P 2011 Mitochondrial DNA data indicate an introduction through Mainland Southeast Asia for Australian dingoes and Polynesian domestic dogs. Proc. R. Soc. B 279, 967–974. (doi:10.1098/rspb.2011.1395) 10.1098/rspb.2011.1395 PMC3259930 21900326

[13] Roullier C, Benoit L, McKey DB, Lebot V 2013 Historical collections reveal patterns of diffusion of sweet potato in Oceania obscured by modern plant movements and recombination. Proc. Natl Acad. Sci. USA 110, 2205–2210. (doi:10.1073/pnas.1211049110) 23341603 10.1073/pnas.1211049110 PMC3568323

[14] Thomson VA et al. 2014 Using ancient DNA to study the origins and dispersal of ancestral Polynesian chickens across the Pacific. Proc. Natl Acad. Sci. USA 111, 4826–4831. (doi:10.1073/pnas.1320412111) 24639505 10.1073/pnas.1320412111 PMC3977275

[15] Greig K, Boocock J, Prost S, Horsburgh KA, Jacomb C, Walter R, Matisoo-Smith E 2015 Complete mitochondrial genomes of New Zealand's first dogs. PLoS ONE 10, e0138536 (doi:10.1371/journal.pone.0138536) 26444283 10.1371/journal.pone.0138536 PMC4596854

[16] Bay-Petersen J 1984 Competition for resources: the role of pig and dog in the Polynesian agricultural economy. J. Soc. Oceanistes 39, 121–129. (doi:10.3406/jso.1983.2793)

[17] McGlone MS, Anderson AJ, Holdaway RN 1994 An ecological approach to the Polynesian settlement of New Zealand. In The origins of the first New Zealanders (ed. Sutton DG), pp. 136–163. Auckland, New Zealand: Auckland University Press.

[18] Flannery TF 1995 The future eaters: an ecological history of the Australasian lands and people. Chatswood, Australia: Reed Books.

[19] Clark G 1997 Osteology of the Kuri Māori: the prehistoric dog of New Zealand. J. Archaeol. Sci. 24, 113–126. (doi:10.1006/jasc.1995.0098)

[20] Buist AG, Yaldwyn JC 1960 An ‘articulated’ moa leg from an oven excavated at Waingongoro, South Taranaki. J. Polynesian Soc. 69, 76–88.

[21] Trotter MM 1975 Archaeological investigations at Redcliffs, Canterbury, New Zealand. Rec. Canterbury Mus. 9, 189–220.

[22] McGovern-Wilson R, Kirk F, Smith I 1996 Small bird remains. In Shag River mouth: the archaeology of an early southern Māori village (eds Anderson A, Allingham B, Smith I), pp. 223–236. Canberra, Australia: ANH Publications.

[23] Jacomb C 2009 Excavations and chronology at the Redcliffs Flat site, Canterbury, New Zealand. Rec. Canterbury Mus. 23, 17–34.

[24] Hill N, Campbell M 2009 Archaeological monitoring of the Oakura wastewater treatment and reticulation upgrade. *Report to the New Zealand Historic Place Trust and Whangarei District Council*. CFG Heritage: Auckland, New Zealand.

[25] Walter R, Jacomb C, Brooks E 2010 Final report on archaeological excavations at Cooks Cove Z17/311, Tologa Bay, East Coast, North Island. Dunedin, New Zealand: Southern Pacific Archaeological Research.

[26] Scofield RP, Worthy TH, Schlumpf H 2003 What birds were New Zealand's first people eating? Wairau Bar's avian remains re-examined. Rec. Canterbury Mus. 17, 17–35.

[27] Perry GL, Wheeler A, Wood JR, Wilmshurst JM 2014 A high-precision chronology for the rapid extinction of New Zealand moa (Aves, Dinornithiformes). Q. Sci. Rev. 105, 126–135. (doi:10.1016/j.quascirev.2014.09.025)

[28] Tennyson AJD, Martinson P 2006 Extinct birds of New Zealand. Wellington, New Zealand: Te Papa Press.

[29] Worthy TH, Holdaway RN 2002 Lost world of the moa. Christchurch, New Zealand: Canterbury University Press.

[30] Storey AA et al. 2007 Radiocarbon and DNA evidence for a pre-Columbian introduction of Polynesian chickens to Chile. Proc. Natl Acad. Sci. USA 104, 10 335–10 339. (doi:10.1073/pnas.0703993104) 10.1073/pnas.0703993104 PMC1965514 17556540

[31] Storey AA et al. 2008 Pre-Columbian chickens, dates, isotopes, and mtDNA. Proc. Natl Acad. Sci. USA 105, E99 (doi:10.1073/pnas.0807625105) 19033182 10.1073/pnas.0807625105 PMC2596261

[32] Gongora J et al. 2008 Reply to Storey *et al*.: more DNA and dating studies needed for ancient El Arenal-1 chickens. Proc. Natl Acad. Sci. USA 105, E100 (doi:10.1073/pnas.0809681105)

[33] Thomson VA et al. 2014 Reply to Beavan, Bryant, and Storey and Matisoo-Smith: ancestral Polynesian ‘D’ haplotypes reflect authentic Pacific chicken lineages. Proc. Natl Acad. Sci. USA 111, E3585–E3586. (doi:10.1073/pnas.1411566111) 25333086 10.1073/pnas.1411566111 PMC4156704

[34] Beavan N 2014 No evidence for sample contamination or diet offset for pre-Columbian chicken dates from El Arenal. Proc. Natl Acad. Sci. USA 111, E3582 (doi:10.1073/pnas.1410794111) 25122683 10.1073/pnas.1410794111 PMC4156739

[35] Smith IWG 2002 The New Zealand sealing industry: history, archaeology, and heritage management. Wellington, New Zealand: Department of Conservation.

[36] Grady D 1986 Sealers & whalers in New Zealand waters. Auckland, New Zealand: Reed Methuan.

[37] Jacobson HC 1914 Tales of Banks Peninsula. Akaroa, New Zealand: Akaroa Mail Office.

[38] McNab R 1909 Murihiku: a history of the South Island of New Zealand and the islands adjacent and lying to the south, from 1642 to 1835. Wellington, New Zealand: Whitcombe and Tombs Limited.

[39] Palmer JG, Turney C, Hogg A, Hilliam N, Watson M, van Sebille E, Cowie W, Jones R, Petchey F 2014 The discovery of New Zealand's oldest shipwreck—possible evidence of further Dutch exploration of the South Pacific. J. Archaeol. Sci. 42, 435–441. (doi:10.1016/j.jas.2013.11.024)

[40] Beaglehole JC 1961 The journals of Captain James Cook on his voyages of discovery. Volume II, The voyage of the Resolution and Adventure 1772–1775. Cambridge, UK: Cambridge University Press.

[41] O'Shea PP 1970 Captain James Cook R.N., F.R.S. and his numismatic associations. Supp. NZ Numismatic J. 12, 1–51.

[42] Lane P 2009 Captain Cook's exploration medals. reCollections 4. See http://recollections.nma.gov.au/issues/vol_4_no1/notes_and_comments/captain_cooks_exploration_medals#11 (accessed 3 September 2014).

[43] Schaniel WC 2001 European technology and the New Zealand Māori economy: 1769–1840. Soc. Sci. J. 38, 137–146. (doi:10.1016/S0362-3319(00)00118-X)

[44] Anderson A 1998 The welcome of strangers. An ethnohistory of Southern Māori AD 1650–1850. Dunedin, New Zealand: University of Otago Press.

[45] Perry GLW, Wilmshurst JM, McGlone MS 2014 Ecology and long-term history of fire in New Zealand. NZ J. Ecol. 38, 157–176.

[46] McWethy DB et al. 2010 Rapid landscape transformation in South Island, New Zealand, following initial Polynesian settlement. Proc. Natl Acad. Sci. USA 107, 21 343–21 348. (doi:10.1073/pnas.1011801107) 10.1073/pnas.1011801107 PMC3003061 21149690

[47] McGlone MS, Wilmshurst JM 1999 Dating initial Māori environmental impact in New Zealand. Q. Int. 59, 5–16. (doi:10.1016/S1040-6182(98)00067-6)

[48] Finney B 1994 Experimental voyaging and Māori settlement. In The origins of the first New Zealanders (ed. Sutton DG), pp. 52–76. New Zealand: Auckland University Press.

[49] Rolett BV 2002 Voyaging and interaction in ancient East Polynesia. Asian Persp. 41, 182–194. (doi:10.1353/asi.2003.0009)

[50] Hogg AG et al. 2013 SHCal13 Southern Hemisphere calibration, 0–50 000 years cal BP. Radiocarbon 55, 1889–1903. (doi:10.2458/azu_js_rc.55.16783)

[51] Bronk Ramsey C 2008 Deposition models for chronological records. Q. Sci. Rev. 27, 42–60. (doi:10.1016/j.quascirev.2007.01.019)

[52] Leonard JA et al. 2007 Animal DNA in PCR reagents plagues ancient DNA research. J. Archaeol. Sci. 34, 1361–1366. (doi:10.1016/j.jas.2006.10.023)

[53] Bandelt HJ, Forster P, Röhl A 1999 Median-joining networks for inferring intraspecific phylogenies. Mol. Biol. Evol. 16, 37–48. (doi:10.1093/oxfordjournals.molbev.a026036) 10331250 10.1093/oxfordjournals.molbev.a026036

