# Supplementary material for: Origin and timing of New Zealand's earliest domestic chickens: Polynesian commensals or European introductions?
Source: R Soc Open Sci. 2016 Aug 3;3(8):160258. doi: 10.1098/rsos.160258 (PMC5108951; doi:10.1098/rsos.160258)
Supplement: Revised_Supp_Info_no mark up [file rsos160258supp2.pdf]

Supporting information

Site descriptions

Mussel Point, north-eastern South Island (c. 41° 43.3’ S, 174° 13.4’ E). Large numbers of bird bones have been collected from coastal dunes along a ca. 4.5 km section of Marfells Beach from Mussel Point to the intake of Lake Grassmere. However, the exact context of most of these bones are unclear, and it seems likely that they include both naturally deposited bones, and bones eroded from Māori middens [1, 2]. A summary of bird bones from the area was presented by Scarlett [1], who identified the presence of several extinct and regionally extirpated taxa, including four of the currently accepted moa species. The sampled chicken bone was collected from Mussel Point (NZ Archaeological Association site number Q29/1), yet has no other metadata associated with it. However, remains of at least two moa species, *Emeus crassus* and *Euryapteryx gravis* have also been collected at Mussel Point [3, 4], and a Māori midden yielding bones of extinct birds has been noted there [5]. Therefore, despite the unclear context of bones from the site, we felt that the association with bones of moa (moa became extinct within the first 200 years of human settlement [6]) and other pre-European extinct birds (Fig. S1), and the relative proximity of Mussel Point to one of New Zealand’s earliest archaeological sites at Wairau Bar [7], made the bone a suitable candidate for analysis.

Fyffe’s Site, eastern South Island (c. 42° 25.3’ S, 173° 42.5’ E). We sampled a chicken bone collected from the “Fyffe site” (NZ Archaeological Association site number S49/46) at Avoca Point, Kaikoura [8]. A spatially dense complex of archaeological sites at Avoca Point were excavated in the 1980s, and included sites dating from the moa hunting period (pre-1450 AD) right up until the post-European era [9]. Fyffe’s Site includes a complex of different occupation layers, from an early Māori midden through to later occupations by gardeners and whalers [9−11]. The exact context of the chicken bone could not be determined, but associated material from the same collection includes bones of moa and other pre-European extinct birds (Fig. S1).

Redcliffs School Site, Christchurch, eastern South Island (c. 43° 33.6’ S, 172° 44’ E). In 1969, an archaeological excavation of 21.5 m^2 was carried out on land that was to be developed for extending the Redcliffs Primary School grounds, near Christchurch [12]. The excavation uncovered a large (ca. 3 m diameter) oven pit with evidence of multiple use. The stratigraphy consisted of a basal layer of charcoal and burnt stones, overlain by ashy sands, shell and bone middens, and an upper disturbed layer containing both recent and prehistoric remains [12]. The bones recovered from the oven are dominated by New Zealand fur seal (*Arctocephalus forsteri*), spotted shag (*Stictocarbo punctatus*), and Fiordland crested penguin (*Eudyptes pachyrhynchus*), but also include a single South Island adzebill (*Aptornis defossor*) and a large number of moa [12]. A relatively high-proportion of pre-European fauna were present in the deposit (Fig. S1). All archaeological sites on Redcliffs Flat that have been radiocarbon dated have returned ages from the earliest settlement phase (pre- AD1450) [13]. Trotter [12] notes that “Some bird bone (including that of the European domestic fowl *Gallus* and pigeon *Columba*) and moa-eggshell were found in disturbed material present in some places beneath the road but on top of the main occupational deposit”. However, the Canterbury Museum register gives the excavated position of the chicken bone we studied as square B6, upper layer. Associated material from this square/layer included native birds (*Eudyptes* penguin, Spotted shag and Little penguin) – with no mention of sheep or other introduced mammals. The upper layer of the adjacent square (C6) also contained native birds, including stout legged moa, kereru, broad-billed prion, parakeet, grey duck and another chicken bone.

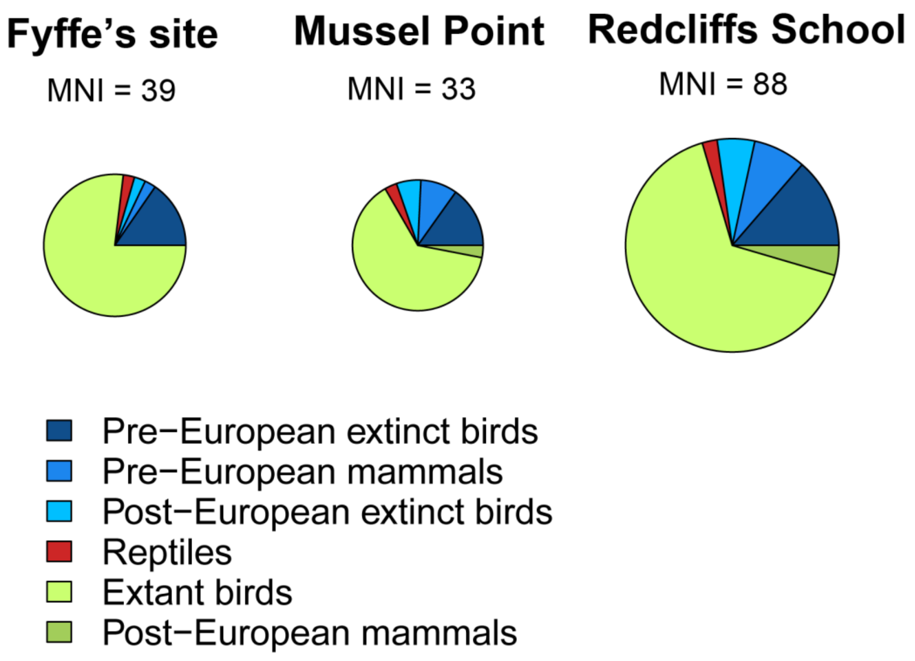

Fig. S1. Relative abundance of terrestrial vertebrate remains (number of individual bones) deposited in Canterbury Museum, Christchurch, New Zealand, from each of the three sites where chicken bones were sampled for this study.

1. Scarlett RJ. 1979 Avifauna and Man. *Birds of a Feather: Osteological and archaeological papers from the South Pacific in honour of R J Scarlett*, ed Anderson A. British Archaeological Reports, International Series 62: Oxford, England, pp. 75−90.

2. Challis AJ. 1991 *A review of archaeological site records for the Canterbury region*. Department of Conservation Sciene and Research Series No. 45: Wellington, New Zealand.

3. Scarlett RJ. 1974 Moa and man in New Zealand. *Notornis* **21**, 1−12.

4. Worthy TH. 1998 A remarkable fossil and archaeological avifauna from Marfells Beach, Lake Grassmere, South Island, New Zealand. *Rec. Cant. Mus.* **12**, 79-176.

5. Millener PR. 1981 The subfossil distribution of the extinct New Zealand coots. *Notornis* **28**, 1−9.

6. Perry GL, Wheeler A, Wood JR, Wilmshurst JM. 2014 A high-precision chronology for the rapid extinction of New Zealand moa (Aves, Dinornithiformes). *Quat. Sci. Rev.* **105**, 126−135.

7. Higham T, Anderson A, Jacomb C. 1999 Dating the First New Zealanders: the chronology of the Wairau Bar. *Antiquity* **73**, 420−427.

8. Trotter MM. 1980 Archaeological investigations at Avoca Point, Kaikoura. *Rec. Cant. Mus.* **9**, 277−288.

9. Trotter M, McCulloch B. 1999 Archaeology of the Fyffe historic area. *Archaeol. NZ* **42**, 44−56.

10. Trotter M, McCulloch B. 1989 *Unearthing New Zealand*. Government Printing Office: Wellington, New Zealand.

11. Trotter M, McCulloch B. 1993 Fyffe's revisited. *Rec. Cant. Mus.* **10**, 73−94.

12. Trotter MM. 1975 Archaeological investigations at Redcliffs, Canterbury, New Zealand. *Rec. Cant. Mus.* **9**, 189−220.

13. Jacomb C. 2009 Excavations and chronology at the Redcliffs Flat site, Canterbury, New Zealand. *Rec. Canterbury Mus.* **23**, 17–34.
